# H_2_–D_2_ Exchange Activity and Electronic Structure of Ag_*x*_Pd_1–*x*_ Alloy Catalysts Spanning Composition Space

**DOI:** 10.1021/acscatal.4c02309

**Published:** 2024-07-08

**Authors:** Nicholas Golio, Irem Sen, Xiaoxiao Yu, Petro Kondratyuk, Andrew J. Gellman

**Affiliations:** ^†^Department of Chemical Engineering and ^‡^W.E. Scott Institute for Energy Innovation, Carnegie Mellon University, Pittsburgh, Pennsylvania 15213, United States

**Keywords:** catalysis, silver, palladium, alloy, H_2_−D_2_ exchange, hydrogen adsorption

## Abstract

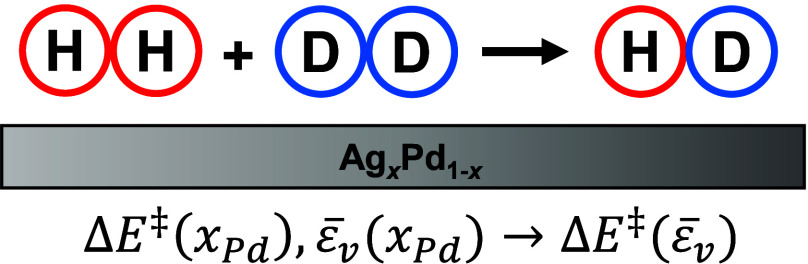

Many computational studies of catalytic surface reaction kinetics have demonstrated the existence of linear scaling relationships between physical descriptors of catalysts and reaction barriers on their surfaces. In this work, the relationship between catalyst activity, electronic structure, and alloy composition was investigated experimentally using a Ag_*x*_Pd_1–*x*_ Composition Spread Alloy Film (CSAF) and a multichannel reactor array that allows measurement of steady-state reaction kinetics at 100 alloy compositions simultaneously. Steady-state H_2_–D_2_ exchange kinetics were measured at atmospheric pressure on Ag_*x*_Pd_1–*x*_ catalysts over a temperature range of 333–593 K and a range of inlet H_2_ and D_2_ partial pressures. X-ray photoelectron spectroscopy (XPS) was used to characterize the CSAF by determining the local surface compositions and the valence band electronic structure at each composition. The valence band photoemission spectra showed that the average energy of the valence band, ε̅_v_, shifts linearly with composition from −6.2 eV for pure Ag to −3.4 eV for pure Pd. At all reaction conditions, the H_2_–D_2_ exchange activity was found to be highest on pure Pd and gradually decreased as the alloy was diluted with Ag until no activity was observed for compositions with *x*_Pd_ < 0.58. Measured H_2_–D_2_ exchange rates across the CSAF were fit using the Dual Subsurface Hydrogen (2H′) mechanism to extract estimates for the activation energy barriers to dissociative adsorption, Δ*E*_ads_^‡^, associative desorption, Δ*E*_des_^‡^, and the surface-to-subsurface diffusion energy, Δ*E*_ss_, as a function of alloy composition, *x*_Pd_. The 2H′ mechanism predicts Δ*E*_ads_^‡^ = 0–10 kJ/mol, Δ*E*_des_^‡^ = 30–65 kJ/mol, and Δ*E*_ss_ = 20–30 kJ/mol for all alloy compositions with *x*_Pd_ ≥ 0.64, including for the pure Pd catalyst (i.e., *x*_Pd_ = 1). For these Pd-rich catalysts, Δ*E*_des_^‡^ and Δ*E*_ss_ appeared to increase by ∼5 kJ/mol with decreasing *x*_Pd_. However, due to the coupling of kinetic parameters in the 2H′ mechanism, we are unable to exclude the possibility that the kinetic parameters predicted when *x*_Pd_ ≥ 0.64 are identical to those predicted for pure Pd. This suggests that H_2_–D_2_ exchange occurs only on bulk-like Pd domains, presumably due to the strong interactions between H_2_ and Pd. In this case, the decrease in catalytic activity with decreasing *x*_Pd_ can be explained by a reduction in the availability of surface Pd at high Ag compositions.

## 1 Introduction

Alloys and multicomponent materials are often used as catalysts because they possess catalytic properties superior to those of their pure components.^1^ The beneficial properties of alloys arise from changes in ligand, strain, and ensemble effects, which influence the physical and electronic characteristics of the material to enhance its catalytic activity, selectivity, and resistance to poisoning.^2,3^ As a result, the catalytic activity of an alloy catalyst can be tuned using its composition.^4,5^ To properly understand how alloying affects the kinetics of chemical reactions, it is necessary to measure reaction rates across a wide range of catalyst compositions.

Acquiring kinetic data that spans alloy catalyst composition space is challenging because preparing and characterizing a large set of single composition samples using conventional methods is costly and time-consuming. Conventional approaches that study one alloy composition at a time drastically limit the feasible number of compositions that can be examined in a single study. In general, optimization of an *n*-component alloy catalyst requires sampling a continuous, *n*-1-dimensional composition space, which is a daunting challenge as the number of components increases. A well-known example of using discrete sampling of catalyst compositions comes from the early 1900s, where Haber and Bosch spent years testing ∼2000 different ammonia synthesis catalysts before identifying Fe as the most active.^6^ The development of high-throughput techniques to address such time-consuming and cost prohibitive experimentation offers enormous opportunities for accelerating catalyst discovery in the future.^7,8^

**Figure 1:**
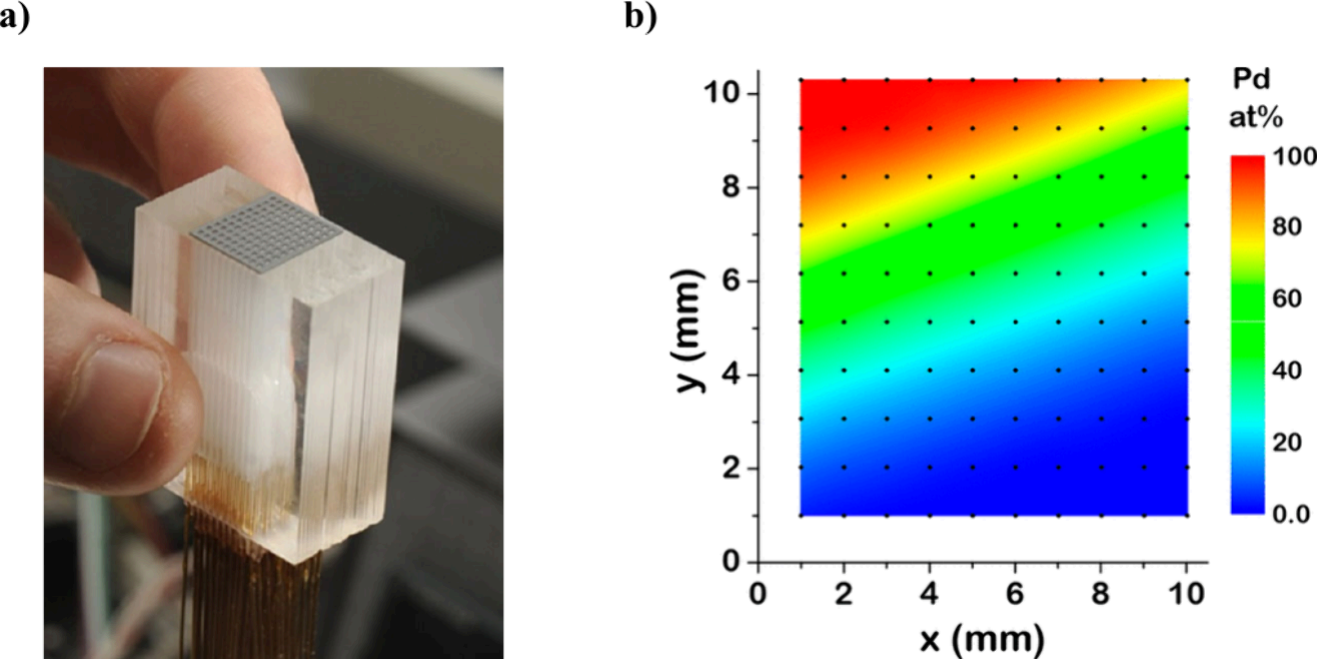
(a) Photograph of the glass microreactor array. The inlet and outlet capillaries are bonded into channels at the bottom of the glass reactor block. A gasket with a 10 × 10 array of holes is positioned on top of the microreactor block to isolate one inlet and outlet channel within each gasket hole. (b) Pd composition measured by X-ray photoelectron spectroscopy (XPS) versus position on the Ag_*x*_Pd_1–*x*_ binary CSAF. The black dots represent measurement points and lie at the center of the corresponding gasket hole. Figure reproduced with permission from ref (49). Copyright 2018 American Chemical Society.

The implementation of high-throughput characterization methods allows measurement of alloy properties at many compositions in a single experiment. These high-throughput techniques require materials libraries, which contain all of the samples of interest, and a set of complementary techniques for measurement of the functional properties (i.e., catalytic activity, electronic structure, local surface composition, etc.). Composition Spread Alloy Films^9,10^ (CSAFs) are one type of materials library that contain the entirety of composition space for a binary or ternary alloy system on a compact substrate. CSAFs are made by depositing thin alloy films onto the substrate such that there is a lateral gradient in the local alloy composition across the substrate. Figure 1b shows the Pd composition versus film position measured by X-ray photoelectron spectroscopy (XPS) for the binary Ag_*x*_Pd_1–*x*_ CSAF used in this work. The composition of the film varies from 100% Ag in the lower right corner of the substrate to 100% Pd in the upper left corner of the substrate, with all possible compositions of Ag_*x*_Pd_1–*x*_ located between these two points. Measuring the catalytic activity of different alloy compositions across the CSAF requires localizing the reaction into well-defined regions that span a narrow composition range. To achieve this spatial resolution, a multichannel microreactor array of our own design^11^ (Figure 1a) isolates 100 different locations (i.e., alloy compositions) on the CSAF allowing for independent measurement of the catalytic activity of 100 alloy catalysts in a single experiment.

Reactions involving H_2_ have a wide range of industrial applications. For example, H_2_ is used as a fuel source in advanced energy systems, such as fuel cells,^12−15^ and is also used in various catalytic hydrogenation reactions.^16−19^ Because H_2_ is often derived from fossil fuels, the separation (i.e., purification) of H_2_ from CO_2_-rich gas mixtures is a critical step in maintaining the supply of hydrogen. Standard methods for H_2_ separation include solvent adsorption, pressure swing adsorption (PSA), and cryogenic distillation, all of which require large capital and energy expenditures.^20,21^ Metal membranes are a promising alternative for purifying H_2_ due to their low operating cost, low energy consumption, and simplicity of operation.^22^ Pd-based membranes are commonly used for H_2_ separation since they can easily dissociate and dissolve H_2_ and have a high permeability for H atoms.^2^ Pd membranes also possess high selectivity to H_2_ transport relative to other gases.^23,24^ It has been shown that Pd and Pd-alloy membranes are stable for several months under H_2_ flow in the temperature range 900–1046 K.^1,3^ However, there are limitations to the use of pure Pd membranes for H_2_ purification due to the lattice mismatch between the α-PdH and β-PdH phases formed when H_2_ absorbs into Pd below its critical point of 571 K and 2 MPa. Transitions between these two hydride phases cause lattice strain which results in the formation of bulk and grain boundary defects.^25^ Ultimately, the embrittlement of Pd caused by the lattice strain can lead to the rupturing of the membrane after repeated pressure and temperature cycling.^26−28^

Alloying Pd with Ag is beneficial since it enhances the mechanical strength of the alloy^26,29,30^ and increases its H atom permeability.^26,27,31^ Addition of 23 at. % Ag to pure Pd was reported to decrease the critical temperature and pressure for the α-PdH to β-PdH transition, as well as increase the H_2_ solubility significantly.^30,31^ The H atom permeability of Ag–Pd alloy membranes was found to be 1.7 times higher than membranes of Cu–Pd and Au–Pd alloys.^31^ Versuchen et al. showed that Ag–Pd alloys have a higher H_2_ solubility than Au–Pd, Cu–Pd, and Pd–Pt alloys, and that this increased solubility is, in part, responsible for the high permeability of H_2_ through Ag–Pd membranes,^32^ relative to other Pd alloys.

The transport of H_2_ through Pd-based membranes involves three principal steps: dissociative adsorption of H_2_ on the upstream side of the membrane surface into H atoms, diffusion of H atoms through the bulk, and finally, associative desorption of H_2_ from the downstream surface.^33,34^ This transport process is known to be limited by the bulk diffusion of H atoms, as manifested by its half-order dependence on H_2_ pressure, *P*_H2_.^35^ Decreasing the membrane thickness increases the order of the H_2_ pressure dependence and increases the permeability of Ag–Pd alloys.^26,30,35^ For ultrathin Ag–Pd alloy films with thicknesses <500 nm, the H_2_ transport was reported to be first-order in *P*_H2_ over the temperature range 373 K – 523 K.^26^ This result indicates that surface reactions, i.e., the H_2_ adsorption and desorption at the upstream and downstream surfaces, dictate the rate of hydrogen transport through ultrathin Ag–Pd membranes.

While the bulk diffusion of H atoms through Pd has been investigated extensively,^36−38^ the surface reactions describing H_2_ adsorption and desorption onto/from Pd-based alloy surfaces are not as well understood. Kinetic analysis of the H_2_–D_2_ exchange reaction (H_2_ + D_2_ → 2HD) can provide valuable information about the adsorption and desorption processes involving H_2_. Hence, quantification of the kinetic parameters governing H_2_–D_2_ exchange over Ag_*x*_Pd_1–*x*_ composition space gives valuable insight into the behavior of Pd-based membranes for hydrogen purification.

Numerous studies of H_2_ on Pd surfaces have shown that it adsorbs with a negligible barrier to dissociation and a high heat of adsorption.^39−42^ On the other hand, H_2_ does not adsorb dissociatively onto Ag single crystal surfaces at room temperature, and its adsorption is predicted to be endothermic.^43−46^ The most straightforward approach for modeling H_2_–D_2_ exchange on Ag_*x*_Pd_1–*x*_ binary alloys involves applying the traditional Langmuir–Hinshelwood (LH) framework, which parametrizes the reaction by just two rate constants: *k*_ads_ for the dissociative adsorption of H_2_ and *k*_des_ for the associative desorption of H_2_. It has been well-documented that the kinetic behavior predicted by the LH mechanism has been found to be inconsistent with several experimental observations. Savara et al. observed that for H_2_–D_2_ exchange on Pd(111) and Pd nanoparticles with *P*_D2_ ≫ *P*_H2_ and high total surface coverage (θ ≅ 1), the reaction order in *P*_D2_ was *n*_D2_ = 0.^47,48^ This deviates from the LH prediction of *n*_D2_^*LH*^ = –1 for *P*_D2_ ≫ *P*_H2_, which arises from the competition between H_2_ and D_2_ for adsorption sites on a nearly saturated surface, θ ≅ 1. Similarly, Sen et al. reported the same deviation from the LH mechanism by observing that the reaction order in *P*_H2_ was *n*_H2_ = 0 when *P*_H2_ ≫ *P*_D2_ and θ ≅ 1 using the same Ag_*x*_Pd_1–*x*_ CSAF presented in this work.^49^

Previously, we explored reaction mechanisms for H_2_–D_2_ exchange that include the presence of subsurface hydrogen, herein denoted by H′, to resolve deviations from the LH framework.^49^ We proposed the Single Subsurface Hydrogen (1H′) mechanism and the Dual Subsurface Hydrogen (2H′) mechanism, which both incorporate the diffusion of surface H and D atoms into and out of the subsurface with an equilibrium constant, *K*_ss_. Fundamentally, the subsurface hydrogen mechanisms require the presence of either one or two adjacent H′ or D′ species in the immediate subsurface to facilitate the adsorption and desorption occurring on the top surface. Analysis of the rate laws derived from these mechanisms under conditions where *P*_H2_ ≫ *P*_D2_ and θ ≅ 1 revealed that the 2H′ mechanism is consistent with *n*_H2_ = 0 observed in the earlier work using this Ag_*x*_Pd_1–*x*_ CSAF.^49^

In a more recent study,^42^ we estimated the kinetic parameters for adsorption, desorption, and surface-to-subsurface diffusion associated with the LH, 1H′, and 2H′ mechanisms by fitting the rate law given by each model to the measured rate of H_2_–D_2_ exchange on the pure Pd region of this Ag_*x*_Pd_1–*x*_ CSAF at different reaction temperatures and inlet pressures of H_2_ and D_2_. By fitting each model to the reaction data, we obtained estimates for the energy barrier to H_2_ adsorption, Δ*E*_ads_^‡^, the energy barrier to H_2_ desorption, Δ*E*_des_^‡^, and the surface-to-subsurface diffusion energy of adsorbed H on Pd, Δ*E*_ss_. The results of kinetic parameter estimation revealed that the LH model predicted that Pd operates in an adsorption-limited regime with low surface coverage and Δ*E*_ads_^‡^ = 51.1 ± 0.6 kJ/mol, which disagrees with the barrierless H_2_ adsorption on Pd previously reported.^39−41^ On the other hand, both models including H′ correctly predicted Δ*E*_ads_^‡^ = 0 kJ/mol, however, only the 2H′ mechanism was also able to match predictions from density functional theory (DFT) for the surface-to-subsurface transition of adsorbed H on Pd(111) and Pd(100).^40^ Thus, kinetic parameter estimation of H_2_–D_2_ exchange on Pd combined with the observed reaction order across Ag_*x*_Pd_1–*x*_ composition space provide strong evidence that the 2H′ mechanism is the most appropriate for describing this reaction.

In this work, we apply the previously established methodology for kinetic parameter estimation and quantification of parameter uncertainty to fit the 2H′ mechanism to the H_2_–D_2_ exchange activity of the Ag_*x*_Pd_1–*x*_ CSAF across composition space. Extracting the kinetic parameters for adsorption (Δ*E*_ads_^‡^), desorption (Δ*E*_des_^‡^), and surface-to-subsurface diffusion (Δ*E*_ss_) versus alloy composition allows us to see the evolution of the energy barriers for Pd activation as it is gradually diluted with Ag. We have also measured the average energy of the valence band, ε̅_*v*_, spanning alloy composition space, *x*_Pd_, allowing us to understand how the electronic structure of the catalyst relates to its measured activity and how both of these quantities are linked to the kinetic parameters describing the reaction. Ultimately, this work characterizes the electronic structure of Ag_*x*_Pd_1–*x*_ alloys, their catalytic activity for H_2_–D_2_ exchange, and the kinetic parameters associated with the 2H′ mechanism for H_2_–D_2_ exchange, giving insight into the complex relationships between catalyst properties within Ag_*x*_Pd_1–*x*_ composition space.

## 2 Experimental Section

Note that the Ag_*x*_Pd_1–*x*_ CSAF used in this work and the rates of H_2_–D_2_ exchange measured across its surface come from a data set that is already used in two previous publications.^42,49^ The first publication used the rates of H_2_–D_2_ exchange measured at 90 compositions of Ag_*x*_Pd_1–*x*_ composition space spanning the range *x* = 0 → 1 to compare the experimentally observed reaction orders in *P*_H2_ with the reaction orders derived from the LH, 1H′, and 2H′ mechanisms.^49^ The second publication used the activity data from the pure Pd catalyst, i.e., *x*_Pd_ = 1, to develop a rigorous approach for estimating the kinetic parameters for each microkinetic model and their associated uncertainties.^42^ The aim of the current work is to apply our procedure for kinetic parameter estimation to the entire data set to estimate Δ*E*_ads_^‡^, Δ*E*_des_^‡^, and Δ*E*_ss_ for the 2H′ mechanism by fitting the rates of H_2_–D_2_ exchange measured across all of Ag_*x*_Pd_1–*x*_ composition space. We have also measured the average valence band energy, ε̅_*v*_, of each catalyst so that its H_2_–D_2_ exchange activity and associated kinetic parameters can be linked to the electronic structure of the alloy. Detailed explanations of CSAF preparation, CSAF characterization, measurement of the H_2_–D_2_ exchange kinetics, and kinetic parameter estimation can be found elsewhere.^42,49^ Below, we briefly summarize the experimental sections pertinent to this work with the addition of a section for the characterization of the electronic structure of the valence band which has not been previously discussed.

### 2.1 CSAF Preparation

The CSAF was prepared by physical vapor deposition of Pd and Ag onto a 14 × 14 × 3 mm^3^ polished Mo substrate (Valley Design Cop.) using a rotatable shadow mask CSAF deposition tool that has been described in detail previously.^49,50^ Mo was chosen as the substrate material because it does not alloy with Ag or Pd at the annealing and reaction temperatures.^3,51−53^ The deposition rates from the Ag and Pd electron beam evaporation sources were controlled independently by the heating power and were calibrated using a quartz crystal microbalance. The film thickness (∼100 nm in this work) was controlled by the deposition time. The position and orientation of the shadow masks resulted in opposing flux gradients of Ag and Pd across the substrate. The CSAF was deposited and then annealed (800 K for 1 h) under ultrahigh vacuum (UHV) conditions, which are sufficient to induce film crystallization.^51,54^

### 2.2 Characterization of CSAF Composition and Electronic Structure

X-ray photoelectron spectroscopy (XPS) of the Ag_*x*_Pd_1–*x*_ CSAF was performed in a ThetaProbe instrument (Thermo-Fisher Scientific Inc.) to map the local composition across the sample surface as described previously.^49^ The CSAF was moved on an automated stage in the ThetaProbe allowing analysis at a grid of predetermined points across the center of the sample. Spatially resolved maps of the Ag 3*d*_*5/2*_ and Pd 3*d*_*3/2*_ XP spectra were obtained by lateral translation of the CSAF with its surface plane intersecting the source-analyzer focal point. The X-ray spot size was ∼200 μm in diameter and the pass energy of the hemispherical energy analyzer was set to 40 eV. Atomic fractions of the components were estimated using the Avantage Data System software package, which contains a library of the binding energies and the relative intensities of XPS peaks for pure metals. These were used to identify the components and their compositions from the measured spectra. The analysis was performed using smart background subtraction and fitting of the characteristic XPS peaks for Ag 3*d*_*5/2*_ and Pd 3*d*_*3/2*_ was done with curves having a 30% Gaussian/Lorentzian shape.

XPS was also used to map the valence band (*v*-band) electronic structure of the CSAF as a function of its composition. The density of filled states was mapped as a function of binding energy with respect to the Fermi level, ε_*F*_. The Fermi level was determined by fitting a complementary error function to the valence band spectra and finding the intersection of a line through its inflection point with the background signal. The value of the binding energy at this intersection represents ε_*F*_ and became the new zero point for the energy scale. The *v*-band spectra were collected at binding energies in the range 0 to 25 eV with an analyzer pass energy of 40 V. The XPS-derived average energy of the filled *v*-band, ε̅_*v*_, was calculated from the smart background subtracted *v*-band spectra obtained from the CSAF.

### 2.3 Measurement of H_2_–D_2_ Exchange Kinetics across the CSAF

The H_2_–D_2_ exchange activity of the Ag_*x*_Pd_1–*x*_ CSAF was measured at 90 different alloy compositions^49^ using a high-throughput 100-channel microreactor array which has been described in detail elsewhere.^11^ In that study, only 90 channels of the reactor were in use because the inlet flow to 1 row of 10 reactors was blocked. Hence, only 90 different catalyst compositions were studied across the Ag_*x*_Pd_1–*x*_ CSAF. Reactant mixtures of H_2_, D_2_, and Ar were delivered continuously to 90 isolated regions of the Ag_*x*_Pd_1–*x*_ CSAF surface, and products were continuously withdrawn from each region for analysis using a quadrupole mass spectrometer (Extrel).

**Figure 2:**
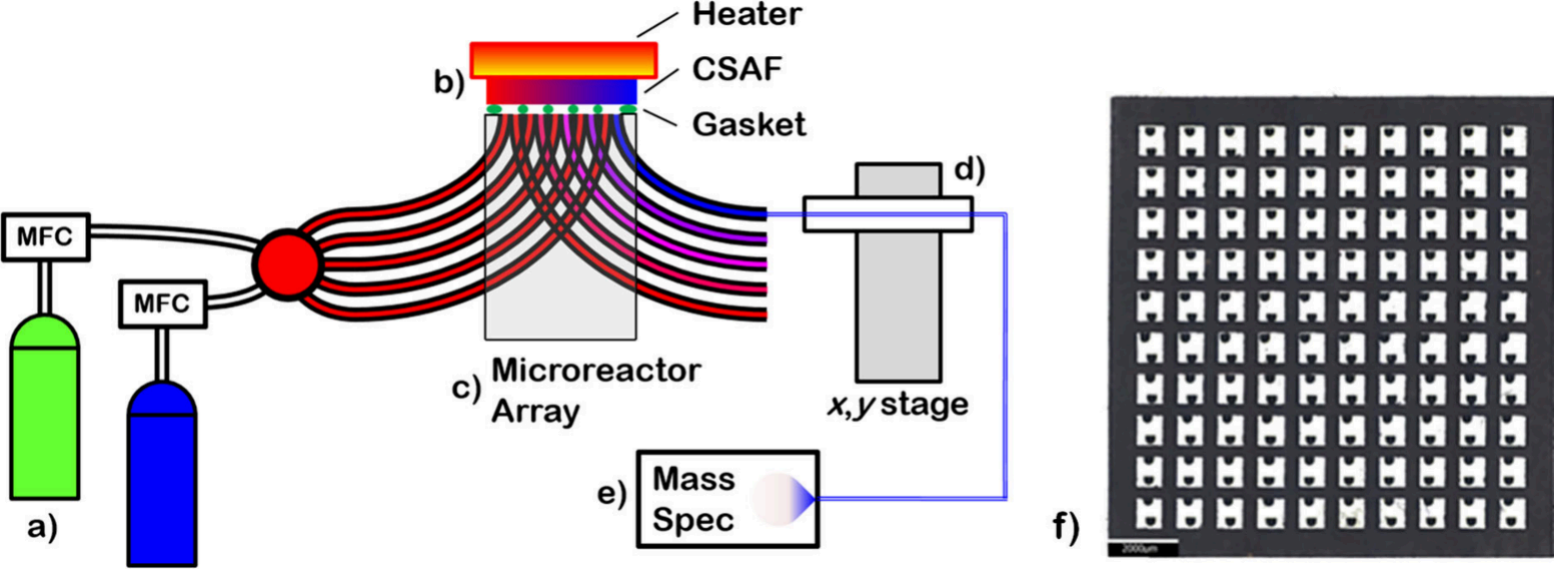
Schematic diagram of the microreactor system. (a) Gas cylinders and mass flow controllers create the feed gas mixture. (b) A high temperature elastomer gasket is used between the CSAF and the microreactor to isolate reaction volumes. A heater is placed on top of the CSAF and a thermocouple is attached to the edge of the Mo substrate. (c) The glass microreactor array with a simplified version of the capillary network showing five inlet–outlet capillary pairs delivering the gas mixture through the system. (d) Automated sampling stage for the product gas streams. Two stepper motors move the sampling capillary in the x and y directions, allowing the sampling arm to be sequentially inserted into and withdrawn from each channel for measurement of the product gas compositions. (e) A quadrupole mass spectrometer performs the analysis of the product gas compositions. (f) An enlarged front view photograph of the gasket represented by the green circles in (b) showing the 10 × 10 array of square holes enclosing each reactor volume. The two dots inside each gasket hole correspond to a pair of inlet and outlet capillary channels which deliver the reaction mixture to and from the CSAF surface.

A schematic representation of the experimental system is shown in Figure 2. The reactant gas mixture is delivered to the microreactor via 360 μm polyimide-coated quartz capillaries (Polymicro Technologies) with the flow rates controlled by mass flow controllers (Aalborg Instruments, GFC-17). The inlet flow is then distributed equally to all 90 microreactor channels. At the surface of the CSAF, pairs of inlet and outlet channels are isolated from their neighbors by a Kalrez 7075 (Dupont) elastomer gasket which has a 10 × 10 array of 700 μm × 900 μm holes (Figure 2f). Gas flows into and out of each reactor volume via a pair of inlet and outlet channels etched into the glass microreactor block. A heater was placed on the backside of the CSAF and the temperature was monitored by a thermocouple that was spot-welded to the edge of the Mo substrate. The product gas streams from each of the 90 independent microreactors were measured using a 10 μm sampling capillary mounted on an automated stage. The capillary was sequentially inserted into each outlet capillary to transport the product gas mixture to the quadrupole mass spectrometer for analysis of its composition. Sampling the product gas mixture in each outlet channel took approximately 7 s, during which time the sampling capillary moved into the channel, waited for steady flow, and then delivered the gas to the mass spectrometer to measure the signal intensity at several values of *m*/*z*. Hence, sampling the reaction products from all 90 microreactor channels (i.e., all Ag_*x*_Pd_1–*x*_ compositions) required ∼12 min.

The H_2_–D_2_ exchange activity of the Ag_*x*_Pd_1–*x*_ alloys on the CSAF was measured at atmospheric pressure and over a range of temperatures from 333 K to 593 K. The range of H_2_ inlet partial pressures was *P*_H2_^in^ = 23 Torr −230 Torr and the range of D_2_ inlet partial pressures was *P*_D2_^in^ = 0.23 Torr −230 Torr with a balance of Ar to keep the total flow rate constant. The temperature was increased from 333 K to 593 K in 20 K increments, and the reaction was allowed to reach steady-state by waiting 4 min at each temperature before analyzing the product gas mixture from each of the reactor channels. In total, 14 different inlet partial pressure combinations of *P*_H2_^in^ and *P*_D2_^in^ were tested at 14 different reaction temperatures for a data set consisting of 196 points for each Ag_*x*_Pd_1–*x*_ catalyst composition. The composition of reaction products was calculated by assuming that the mass spectrometer signals at *m*/*z* = 2, 3, and 4 amu obtained from the product gas samples were proportional to the H_2_, HD, and D_2_ partial pressures, respectively. Baseline signals for 0% conversion at *m*/*z* = 2, 3, and 4 amu were collected by bypassing the CSAF altogether and sampling the feed gas mixture directly. The data set collected from these measurements consists of the HD flow rate exiting each of the reactor channels measured over a range of catalyst compositions, temperatures, and inlet partial pressures, *F*_HD_^exp^ (*x*_Pd_,*T*;*P*_H2_^in^,*P*_D2_^in^).

### 2.4 Kinetic Parameter Estimation and Quantification of Parameter Uncertainty for the 2H′ Mechanism for H_2_–D_2_ Exchange

Kinetic parameters for the 2H′ mechanism for H_2_–D_2_ exchange were estimated across the Ag_*x*_Pd_1–*x*_ CSAF using an optimization routine that has been described in detail elsewhere.^42^ Briefly, the fitting was performed by decoupling all Arrhenius rate constants by fixing the pre-exponential factors to values calculated from transition state theory: *v*_ads_ = 10^2^ mol/m^2^/s/Torr for dissociative adsorption, *v*_des_ = 10^6^ mol/m^2^/s for associative desorption, and *v*_ss_ = 10^0^ for surface-to-subsurface diffusion.^55^ Note that the same pre-exponential factors were used when fitting the reaction data from all active Ag_*x*_Pd_1–*x*_ alloys. While this might have introduced some systematic error into the fits, it was necessary to reduce the degrees of freedom and make the fitting problem tractable given the size of the data set. The MATLAB minimization tool *fmincon* was used to fit the rate law for *F*_HD_ production given by the 2H′ mechanism to the set of *F*_HD_^exp^ at each alloy composition by generating 100 initial guesses for the adsorption energy barrier, Δ*E*_ads_^‡^, desorption energy barrier, Δ*E*_des_^‡^, and surface-to-subsurface diffusion energy, Δ*E*_ss_, within the range 0–100 kJ/mol. The solver minimized the relative sum of squared errors (eq 1) over all 196 data points by varying Δ*E*_ads_^‡^, Δ*E*_des_^‡^, and Δ*E*_ss_ within their respective search spaces until the error between the experimental HD flow rate (*F*_HD_^exp^) and model HD flow rate (*F*_HD_^model^) was minimized. The set of parameter values for Δ*E*_ads_^‡^, Δ*E*_des_^‡^, and Δ*E*_ss_ that yielded the lowest value of χ^2^ among the 100 optimizations was chosen as the optimal solution. Note that the constraint Δ*E*_ss_ > 0 kJ/mol was imposed due to the presence of two mathematically indistinguishable solutions arising from the fact that identical values of *F*_HD_^model^ are obtained when the coverage on the surface, θ, and the coverage in the subsurface, *θ’*, are interchanged in the expression for the flow rate derived from the 2H′ mechanism. In this case, DFT calculations show that the transition of H between the surface and the subsurface should be endothermic for Pd,^40,45^ which is consistent with the constraint that Δ*E*_ss_ > 0.
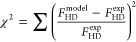
1

The 95% confidence region around the optimal solution (Δ*E*_ads_^‡^, Δ*E*_des_^‡^, Δ*E*_ss_) was determined using the Hessian matrix returned by the solver, which is comprised of all the second derivates of the objective function, χ^2^ (eq 1), with respect to the fitting parameters at the global minimum, χ_min_^2^. This curvature around χ_min_^2^ within parameter space allows construction of a hyper-ellipsoid bounding the region in which the optimal parameter values (Δ*E*_ads_^‡^, Δ*E*_des_^‡^, Δ*E*_ss_) can be found with 95% confidence. The 3D hyper-ellipsoid for the 2H′ mechanism can be visualized in any 2D plane by taking the cross section of the hyper-ellipsoid when the third parameter is fixed to its value at the global minimum. Since χ^2^ exhibits nonquadratic behavior within parameter space, we performed a Taylor expansion from the global minimum at χ_min_^2^ to any point on the 95% confidence ellipsoid to identify the constant contour level within which all combinations of kinetic parameters produce an equivalent fit to the data within the limit of 95% confidence. The extrema of these contour levels provide conservative estimates for the uncertainty of the kinetic parameters found at the global minimum (Δ*E*_ads_^‡^, Δ*E*_des_^‡^, Δ*E*_ss_). It is important to note that for the 2H′ mechanism, the 95% confidence limits for each parameter are given in two separate 2D plots, each representing a different cross section through the 3D hyper-ellipsoid. When these 95% confidence limits differ, we have combined the ranges to encompass all parameter values from both cross sections so that the estimates of the 95% confidence regions include the broadest set of values. An example is given in the Supporting Information for the determination of the 95% confidence limits for Ag_0.1_Pd_0.9_. Figure S1 shows how ln(χ^2^) varies within parameter space near the global minimum and how each elliptical cross section is transformed into a usable uncertainty range.

## 3 Results

### 3.1 Characterization of CSAF Composition

The near surface composition of the Ag_*x*_Pd_1–*x*_ CSAF was mapped by X-ray photoelectron spectroscopy (XPS) and the composition map corresponding to the measurements of catalytic reaction kinetics is reproduced from Sen et al. in Figure 1b.^49^ In the region sampled by the microreactor array, the Ag_*x*_Pd_1–*x*_ CSAF spanned the composition range *x* = 0 → 1.

### 3.2 Characterization of CSAF Electronic Structure

**Figure 3:**
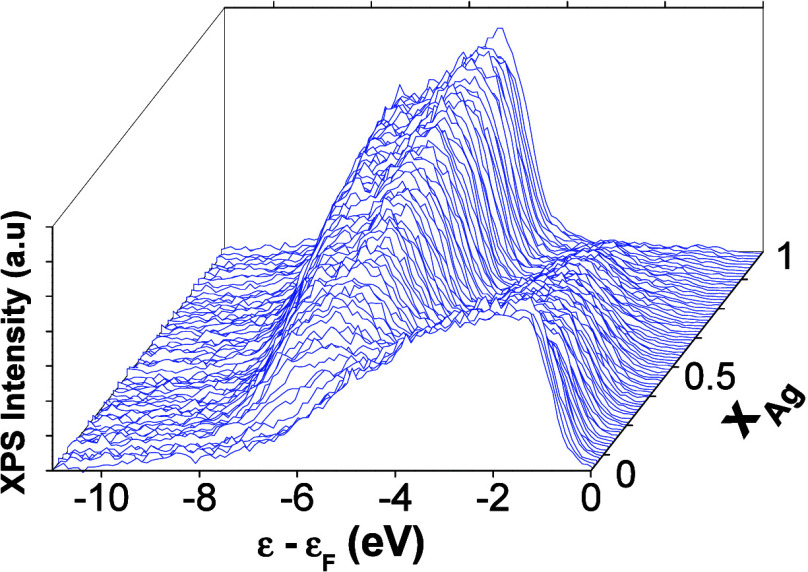
Background subtracted valence band spectra measured by X-ray photoelectron spectroscopy at 169 different Ag_*x*_Pd_1–*x*_ compositions spanning the entire composition space, *x*_Ag_ = 0 → 1.

XPS was also used to map the valence band electronic structure of the Ag_*x*_Pd_1–*x*_ CSAF across composition space. Figure 3 shows the background subtracted *v*-band spectra mapped at 169 discrete locations across the alloy film. The spectra are plotted as functions of energy with respect to the Fermi level, which is located at zero on the energy scale. Figure 3 shows that the density of states shifts toward the Fermi level as the Pd composition increases.

The XPS-derived average energy of the filled *v*-band, ε̅_*v*_ (*x*_Ag_), was calculated in eq 2 from the background subtracted *v*-band spectra (Figure 3) obtained from the Ag_*x*_Pd_1–*x*_ CSAF.


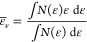
2

The quantity ε is the energy of filled states in the *v*-band relative to the Fermi level and *N*(ε) is the density of filled states, which is taken to be proportional to the intensity of the *v*-band spectrum at a given energy. The XPS-derived average energies of the filled *v*-band, ε̅_*v*_ (*x*_Ag_), were calculated for each alloy composition over the binding energy range −10 to 0 eV. It should be noted that XP spectra only measure the density of filled states, and not the density of empty states. However, the valence bands in Ag_*x*_Pd_1–*x*_ alloys are dominated by filled states since the Ag *d*-band is full and the Pd *d*-band has nine electrons. In this case, the value of ε̅_*v*_ (*x*_Ag_) obtained from the XP spectra only slightly underestimates the average energy of the entire valence band.

**Figure 4:**
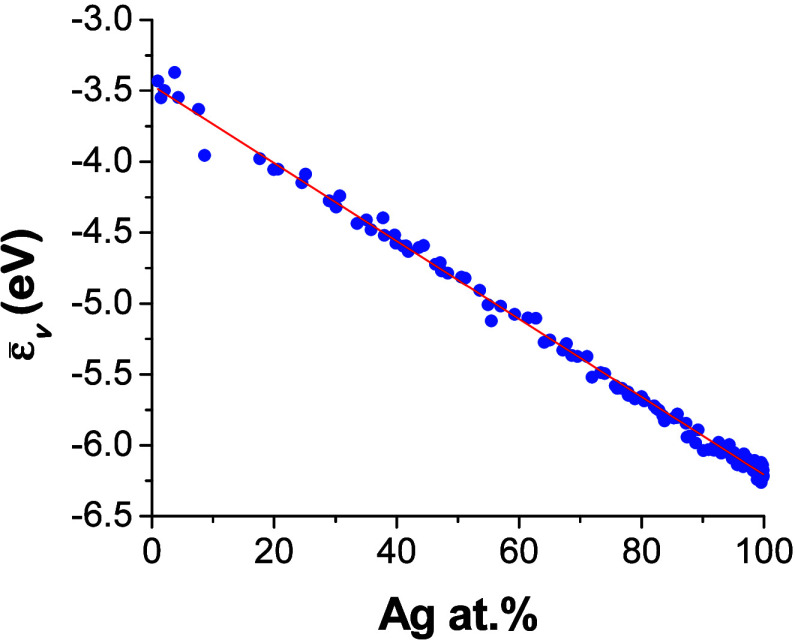
XPS-derived average energy of the filled *v*-band, ε̅_*v*_, relative to the Fermi level of the Ag_*x*_Pd_1–*x*_ CSAF versus Ag at. % composition.

The Ag_*x*_Pd_1–*x*_*v*-band energies shift linearly away from the Fermi level with increasing Ag content (Figure 4). This result is in good agreement with the observation from Figure 3 that the contributions to the *v*-band from Ag and Pd do not mix. Estimates of *d*-band center values for pure Ag and Pd are available in the literature, having been obtained from electronic structure calculations using DFT. The range of *d*-band energies reported for Ag is ε̅_*d*_ = −5.28 eV to −4.28 eV,^56−58^ roughly 1 eV high than our measurement of ε̅_*v*_^Ag^ = −6.2 eV. For Pd, ε̅_*d*_ = −2.64 eV to −2.02 eV,^56−60^ which is also ∼1 eV higher than our measurement of ε̅_*v*_^Pd^ = −3.4 eV. The ∼1 eV offset between the literature values of the *d*-band center and our measurements may arise from the specific approach used to extract ε̅_*v*_ from the valence band spectra (Figure 3). For a given density of states, the choice of the upper and lower integration limits can influence the predicted values of the *d*-band center. Another reason for the offset may be an incomplete background subtraction of secondary electrons contributing to the XP spectra at high binding energies. Finally, the offset may also arise from the fact that our experimental measurements include photoemission from the *s*- and *p*- bands, not just the *d*-band. These issues aside, the offset is not critical to our use of the XPS-derived estimates of ε̅_*v*_ (*x*_Ag_) as we are principally interested in correlating the composition dependence of surface reaction barriers with the composition dependence of ε̅_*v*_ (*x*_Ag_) (i.e., the slope of ε̅_*v*_ (*x*_Ag_) is more important than its absolute value). The fact that both XPS and DFT give the same magnitude of the shift in ε̅_*v*_ with Ag_*x*_Pd_1–*x*_ composition is sufficient.

### 3.3 H_2_–D_2_ Exchange Activity across the Ag_*x*_Pd_1–*x*_ CSAF

**Figure 5:**
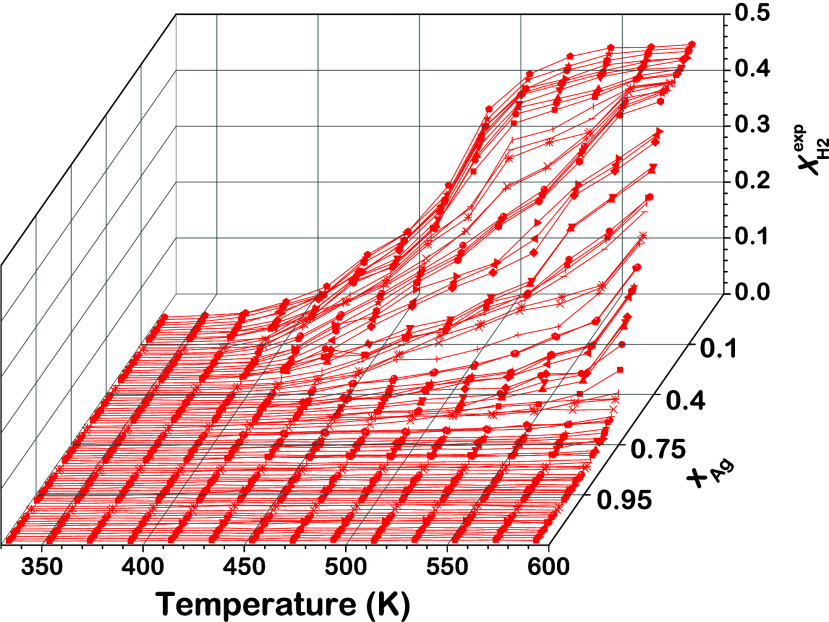
Experimental H_2_ conversion, *X*_H2_^exp^, versus temperature measured across Ag_*x*_Pd_1–*x*_ composition space for a flow consisting of 0.10 mL/min H_2_, 0.10 mL/min D_2_, and 0.13 mL/min Ar in each channel at a total pressure of 760 Torr. For all 90 Ag_*x*_Pd_1–*x*_ compositions, conversion increases with temperature and decreases with Ag content, *x*_Ag_. Figure reproduced with permission from ref (49). Copyright 2018 American Chemical Society.

Catalytic H_2_–D_2_ exchange over the Ag_*x*_Pd_1–*x*_ CSAF was performed by Sen et al. by feeding H_2_, D_2_, and Ar mixtures into the microreactor array at a constant temperature, inlet partial pressure, and flow rate, while monitoring the product gas composition by mass spectrometry.^49^ A table containing the 14 different inlet partial pressure combinations of H_2_ and D_2_ used in the collection of these data sets can be found in the Supporting Information of our previous publication.^49^ The hydrogen conversion, *X*_H2_^exp^, measured at one inlet flow condition (0.10 mL/min H_2_, 0.10 mL/min D_2_, and 0.13 mL/min Ar per channel) plotted versus the reaction temperature, *T*, and alloy composition, *x*_Ag_, is reproduced from Sen et al. in Figure 5.^49^ The hydrogen conversion as a function of *T* and *x*_Ag_ for all inlet flow conditions can be found in the Supporting Information of our previous publication.^49^

**Figure 6:**
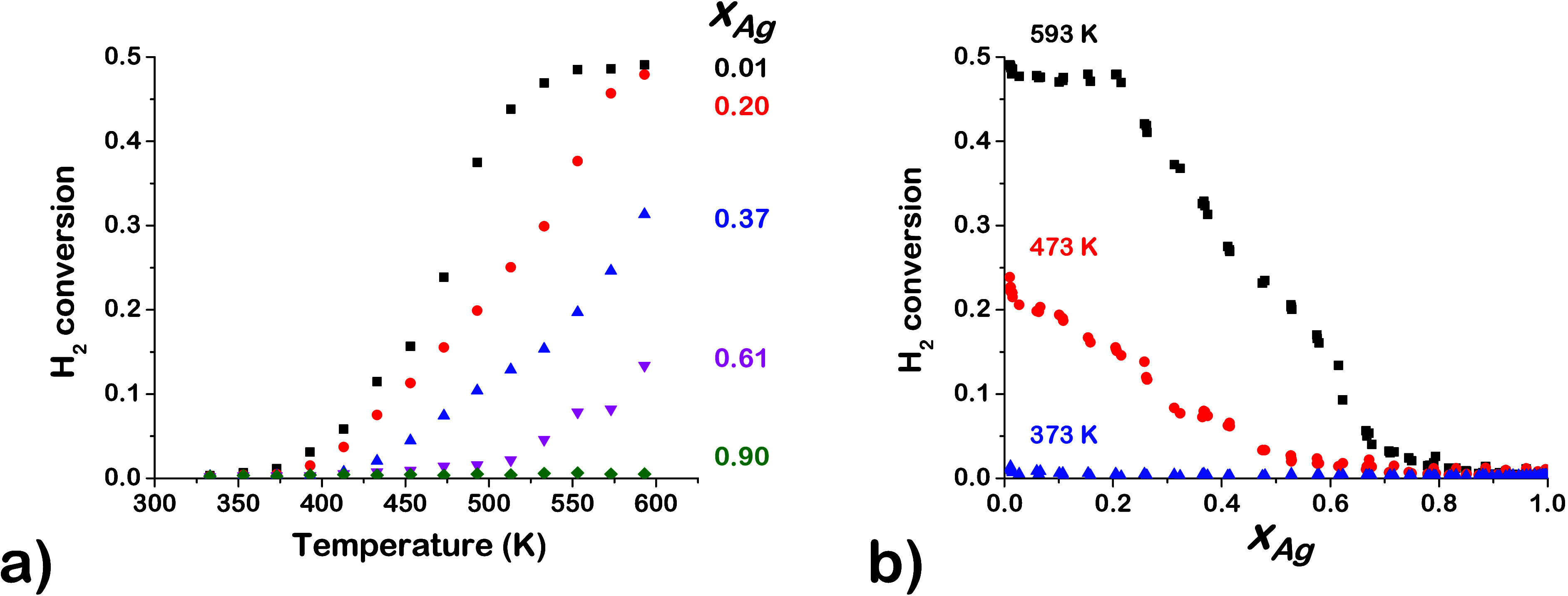
H_2_ conversion for a flow consisting of 0.10 mL/min H_2_, 0.10 mL/min D_2_, and 0.13 mL/min Ar per channel: (a) H_2_ conversion versus reaction temperature, *T*, at five different Ag compositions, *x*_Ag_. H_2_ conversion increases with temperature and decreases with *x*_Ag_. (b) H_2_ conversion versus *x*_Ag_ at three different reaction temperatures. Equilibrium conversion is only achieved when *x*_Ag_ ≤ 0.2 at high *T*.

Conversion versus temperature curves, *X*_H2_ (*T*), were obtained for all inlet flow conditions at each of the 90 different Ag_*x*_Pd_1–*x*_ alloy compositions. Examples are shown in Figure 6a for an inlet flow rate of 0.10 mL/min H_2_, 0.10 mL/min D_2_, and 0.13 mL/min Ar per channel for five different alloy compositions. Note that equilibrium at 593 K corresponds to a conversion of *X*_H2_ = 0.5 when the inlet is composed of equimolar amounts H_2_ and D_2_. Across the CSAF, the H_2_–D_2_ exchange activity increases with increasing reaction temperature, *T*, and decreasing Ag content, *x*_Ag_. Alloy compositions with *x*_Ag_ > 0.62 showed no catalytic activity at any temperature or inlet partial pressure. At *T* ≤ 373 K, the conversion was *X*_H2_ ≈ 0 at all alloy compositions. Equilibrium conversion was reached at the Pd-rich end of the CSAF for *T* > 513 K. As the Pd content decreases, the reaction temperature needed to reach equilibrium conversion increases. For Pd compositions *x*_Pd_ < 0.80, equilibrium was not achieved even at the highest reaction temperature, *T* = 593 K. Figure 6b shows *X*_H2_ versus *x*_Ag_ for the same flow condition as in Figure 6a at three different temperatures. At *T* = 593 K, the activity increases as the Pd content of the alloy increases and reaches the equilibrium conversion for *x*_Ag_ ≤ 0.20. At all inlet flow conditions, H_2_ conversion was found to increase with temperature and Pd content, *x*_Pd_ (Figure 5).

### 3.4 Kinetic Parameters for H_2_–D_2_ Exchange Using the 2H′ Mechanism

Kinetic parameters for H_2_–D_2_ exchange were estimated across Ag_*x*_Pd_1–*x*_ alloy composition space using the 2H′ mechanism. Only a subset of the entire composition range was used for parameter estimation since alloys with *x*_Pd_ < 0.58 showed negligible catalytic activity at all reaction temperatures and inlet flow conditions (Figure 5). In addition, the kinetic parameter estimates at 7 alloy compositions with *x*_Pd_ ≥ 0.58 were discarded due to the fact that the parameters predicted by the 2H′ model were incapable of appropriately fitting the data. As shown in Figure S2, the optimal solutions within parameter space yielded poor fits to the data and the values of χ^2^ associated with these fits were significantly higher than for the rest of the data set shown in Figure S2a. As a result, the kinetic parameters associated with these 7 alloy compositions were determined to be outliers and were removed from consideration for subsequent analysis. Thus, experimental data for 24 alloy compositions spanning *x*_Pd_ = 0.58 → 1, 14 reaction temperatures from *T* = 333 K – 593 K, and 14 inlet flow conditions were used to estimate the kinetic parameters for H_2_–D_2_ exchange as a function of alloy composition, i.e., Δ*E*_ads_^‡^ (*x*_Pd_), Δ*E*_des_^‡^ (*x*_Pd_), Δ*E*_ss_ (*x*_Pd_).

**Figure 7:**
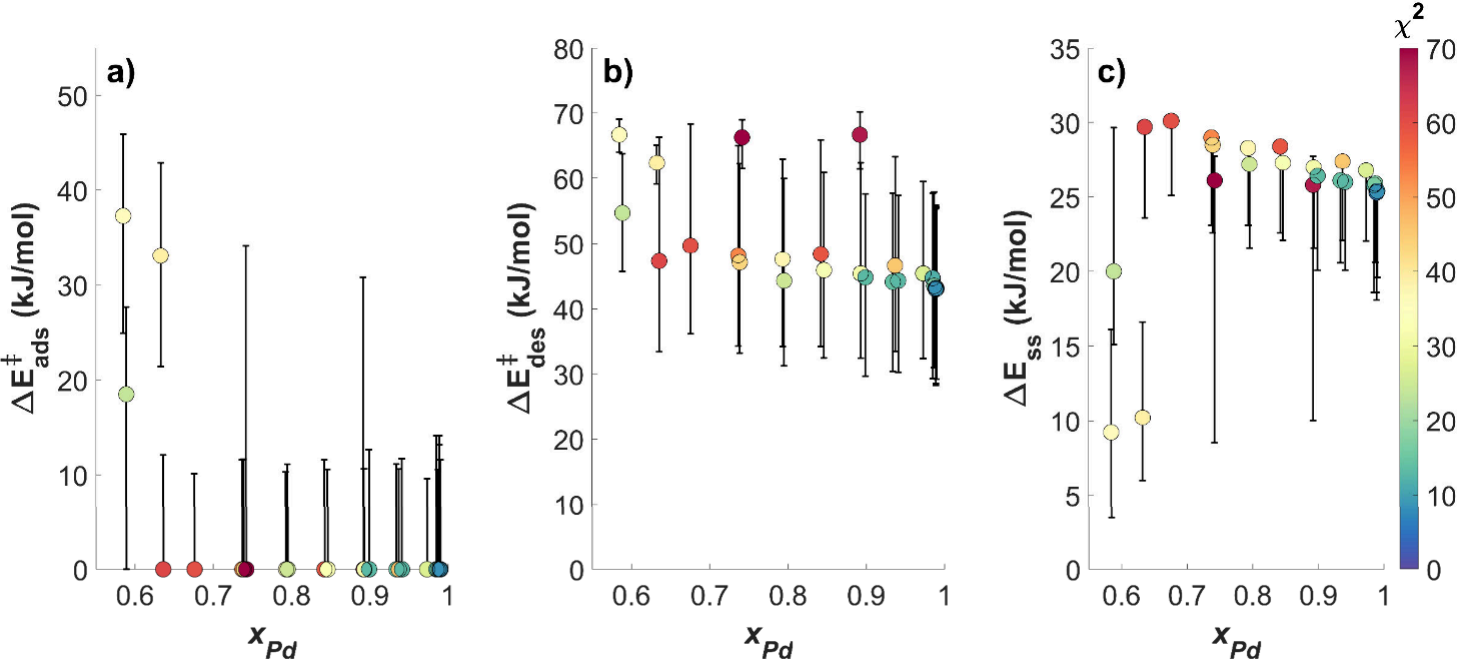
Kinetic parameters for H_2_–D_2_ exchange predicted by the 2H′ mechanism, (a) Δ*E*_ads_^‡^, (b) Δ*E*_des_^‡^, and (c) Δ*E*_ss_, versus *x*_Pd_. The value of χ^2^ for the fit at each value of *x*_Pd_ is indicated by the color of the data point and corresponds to the color scale at the right of the figure. The trend in the kinetic parameters with alloy composition changes based upon whether the data points lie above or below *x*_Pd_ = 0.64. When *x*_Pd_ ≥ 0.64, Δ*E*_ads_^‡^ remains 0 kJ/mol (as for pure Pd), whereas the global minima for Δ*E*_des_^‡^ and Δ*E*_ss_ appear to increase with decreasing *x*_Pd_. It is important to note that the trend in the kinetic parameters cannot be determined conclusively due to the degree of coupling between Δ*E*_ads_^‡^, Δ*E*_des_^‡^, and Δ*E*_ss_ in the 2H′ mechanism. The error bars representing the 95% confidence limits around the global minima were chosen to include all regions of parameter space that are capable of achieving equivalent fits to the data set. Since the error bars for *x*_Pd_ ≥ 0.64 all have some degree of overlap, we are unable to exclude the possibility that the kinetic parameters predicted for *x*_Pd_ ≥ 0.64 are identical to those predicted for pure Pd (*x*_Pd_ = 1) within the limit of 95% confidence. Conservatively reporting the kinetic parameters for Ag_*x*_Pd_1–*x*_ catalysts with *x*_Pd_ ≥ 0.64, therefore, results in Δ*E*_ads_^‡^ = 0–10 kJ/mol, Δ*E*_des_^‡^ = 30–65 kJ/mol, and Δ*E*_ss_ = 20–30 kJ/mol. A sudden change in the kinetic parameters is observed for alloy compositions with *x*_Pd_ < 0.64, where Δ*E*_ads_^‡^ increases to between 15–45 kJ/mol, the range for Δ*E*_des_^‡^ narrows to between 50–70 kJ/mol, and Δ*E*_ss_ decreases to between 5–20 kJ/mol. The change in the kinetic parameters for alloy compositions with *x*_Pd_ < 0.64 presumably results from Ag enrichment at the surface of the alloy, beyond which the catalytic activity for H_2_–D_2_ exchange is negligible. Note that seven alloy compositions within the range 0.58 ≤ *x*_Pd_ ≤ 1 are omitted from this figure since the kinetic parameters found by the solver did not yield an appropriate fit to the experimental data (see Figure S2). Exclusion of these seven data points was justified by the fact that the values of χ^2^ associated with these fits were much higher (>1.5 × χ_avg_^2^) than those found for the rest of the data set and were therefore considered to be outliers.

Figure 7 shows the global minima for Δ*E*_ads_^‡^, Δ*E*_des_^‡^, and Δ*E*_ss_ versus *x*_Pd_ as found by the solver when fitting the 2H′ mechanism to the H_2_–D_2_ exchange activity of the Ag_*x*_Pd_1–*x*_ catalysts. The trend in the fitted kinetic parameters depends on whether the data points lie above or below *x*_Pd_ = 0.64, which appears to be a threshold in alloy composition indicating a change in catalyst behavior. When *x*_Pd_ ≥ 0.64, Δ*E*_ads_^‡^ remains 0 kJ/mol (as for pure Pd), whereas the global minima for Δ*E*_des_^‡^ and Δ*E*_ss_ start at 43 kJ/mol and 25 kJ/mol, respectively, and appear to increase by ∼5 kJ/mol with decreasing *x*_Pd_. It is important to note that due to the degree of coupling between Δ*E*_ads_^‡^, Δ*E*_des_^‡^, and Δ*E*_ss_ in the 2H′ mechanism, the exact trend in the kinetic parameters for alloy compositions with *x*_Pd_ ≥ 0.64 cannot be conclusively confirmed. The error bars in Figure 7 representing the 95% confidence intervals around the global minima were chosen to include all regions of parameter space that are capable of achieving equivalent fits to the data set. This is clearly shown in Figure S1 where the landscape of ln(χ^2^) inside the constant contour level used to define the error bars is relatively flat across broad regions of parameter space. Consequently, the overlapping error bars for Δ*E*_des_^‡^ and Δ*E*_ss_ when *x*_Pd_ ≥ 0.64 prevent us from excluding the possibility that the kinetic parameters predicted for *x*_Pd_ ≥ 0.64 are identical to those for pure Pd (*x*_Pd_ = 1) within the limit of 95% confidence. In this case, conservatively reporting the kinetic parameters for Ag_*x*_Pd_1–*x*_ catalysts with *x*_Pd_ ≥ 0.64 results in Δ*E*_ads_^‡^ = 0–10 kJ/mol, Δ*E*_des_^‡^ = 30–65 kJ/mol, and Δ*E*_ss_ = 20–30 kJ/mol.

On the other side of the composition threshold, for alloys with *x*_Pd_ < 0.64, Δ*E*_ads_^‡^ is predicted to increase to 15–45 kJ/mol, the range for Δ*E*_des_^‡^ narrows to between 50–70 kJ/mol, and Δ*E*_ss_ decreases to between 5–20 kJ/mol. This change in the kinetic parameters presumably results from Ag enrichment at the surface of the alloy when *x*_Pd_ < 0.64, beyond which the activity of the catalysts is negligible. The increased amount of Ag on the top surface creates an appreciable barrier to reaction because the dissociative adsorption of H_2_ onto Ag surfaces is known to be endothermic.^43−46^ A decrease in Δ*E*_ss_ for alloys with *x*_Pd_ < 0.64 implies a decrease in the energy barrier for the surface-to-subsurface transition of adsorbed H and D species. This may suggest that Pd is present in the subsurface of the alloy, just below a top surface that is mostly Ag-enriched. Since interactions between H and Pd in the subsurface are likely more favorable than interactions between H and surface Ag, this leads to a reduction in Δ*E*_ss_ when the surface Ag composition is sufficiently high.

## 4 Discussion

### 4.1 Evaluation of Kinetic Parameters for H_2_–D_2_ Exchange on Ag_*x*_Pd_1–*x*_

Previously, we provided evidence that H_2_–D_2_ exchange on the pure Pd catalyst from this CSAF was most accurately described by the 2H′ mechanism^42,49^ since it predicts Δ*E*_ads_^‡^ ≅ 0 kJ/mol, consistent with numerous studies of H_2_ dissociation on Pd surfaces,^39−41^ and it properly accounts for the zero-order dependence of the reaction rate on *P*_H2_^in^ (i.e., *n*_H2_ = 0) under conditions where *P*_H2_^in^ ≫ *P*_D2_^in^ and θ ≈ 1.^49^ In addition, the 2H′ mechanism’s prediction of Δ*E*_ss_ ≅ 25 kJ/mol is similar to the values of Δ*E*_ss_ = 29 kJ/mol for Pd(111) and Δ*E*_ss_ = 30 kJ/mol for Pd(100) calculated in DFT studies.^40^ Thus, we have strong evidence that the 2H′ mechanism is the most consistent model for describing H_2_–D_2_ exchange kinetics on Pd.

Interestingly, the values of Δ*E*_ads_^‡^, Δ*E*_des_^‡^, and Δ*E*_ss_ predicted by the 2H′ mechanism for all Ag_*x*_Pd_1–*x*_ alloy catalysts with *x*_Pd_ ≥ 0.64 might be identical to those predicted for the pure Pd catalyst (i.e., *x*_Pd_ = 1), as seen in Figure 7. The global minima within parameter space consistently predict Δ*E*_ads_^‡^ = 0 for *x*_Pd_ ≥ 0.64, and while there is a slight trend in Δ*E*_des_^‡^ and Δ*E*_ss_ with alloy composition, the error bars encompassing the regions of 95% confidence almost entirely overlap with the 95% confidence interval for *x*_Pd_ = 1. Due to the uncertainty inherent in our estimation of kinetic parameters, we are unable to discriminate such minor changes (i.e., ≤ 5 kJ/mol) in Δ*E*_ads_^‡^, Δ*E*_des_^‡^, and Δ*E*_ss_ and instead, choose to consider the entire 95% confidence regions bounded by the error bars in Figure 7. While this limits some of the conclusions that can be drawn from the results, the overall trend for how the kinetics of H_2_–D_2_ exchange change with alloy composition can still be observed. Our results suggest that an increase in Δ*E*_ss_ can be expected with decreasing *x*_Pd_ at dilute Ag compositions. This is consistent with the understanding that alloying Ag with Pd will increase the energy barrier for populating the subsurface with H′ species.

On the other hand, taking into account the overlapping 95% confidence limits for Δ*E*_ads_^‡^, Δ*E*_des_^‡^, and Δ*E*_ss_ in Figure 7 requires us to also consider the possibility that the kinetic parameters predicted by the 2H′ mechanism do not vary with alloy composition when *x*_Pd_ ≥ 0.64. In this case, one has to ask what would cause the catalytic activity to drop as *x*_Pd_ decreases from 1 to 0.64. Our observations suggest that Pd bears the entire catalytic load for H_2_–D_2_ exchange, and that Ag merely serves as a diluent. In other words, the kinetic parameters might be invariant with alloy composition over this range because the reaction only occurs on Pd and thus, the behavior resembles that of a pure Pd catalyst provided that Pd is present on the surface in sufficient quantities. The decrease in activity, therefore, arises from the progressive dilution with Ag atoms, which occupy more surface sites as *x*_Pd_ decreases, reducing the effective Pd surface area for H_2_ conversion. When more Ag atoms are present in the alloy and occupy surface sites, the mean size and dispersion of Pd ensembles decrease. As progressively fewer and smaller Pd domains are present in the alloy, this results in decreased H_2_–D_2_ exchange activity. The effect of the size of Pd ensembles on their effectiveness as H_2_–D_2_ exchange catalysts can be examined further using thermodynamic modeling approaches, such as kinetic Monte Carlo simulations, to obtain average surface reaction configurations at various temperatures and alloy compositions.

What is certainly apparent from our results, however, is that the estimated kinetic parameters reflect a change in catalyst behavior at the threshold composition of *x*_Pd_ = 0.64. The kinetic parameters predicted for H_2_–D_2_ exchange when *x*_Pd_ < 0.64 change abruptly from the trend observed for more Pd-rich compositions and are notably different from the values for pure Pd. In particular, there is an increase in the value of Δ*E*_ads_^‡^ accompanied by a decrease in Δ*E*_ss_. Since this change in kinetic parameters occurs right at the threshold in Pd composition below which no catalytic activity is observable (Figure 5), we attribute this difference to the near saturation of Ag at the top surface of the alloy, rendering Pd atoms almost completely inaccessible. To clarify, our interpretation is that the saturation of Ag at the top surface occurs gradually, at the same pace as the incremental replacement of Pd atoms corresponding to the decrease in *x*_Pd_. Although dilution with Ag progressively decreases the H_2_–D_2_ exchange activity (Figure 5), the Pd ensembles present on the surface appear to be large enough to behave as if they were pure Pd (Figure 7). Support for this claim comes from a related work studying Ag-rich Ag_*x*_Pd_1–*x*_ nanoparticles,^61^ which found that large clusters of Pd behaving like pure, bulk-like Pd were present and well-dispersed when *x*_Pd_ ≥ 0.33. Nonetheless, in this work when *x*_Pd_ < 0.64, the change in kinetic parameters captures a difference in catalyst behavior that is not observed in alloys that are more Pd-rich. Perhaps, at this composition threshold, the dissociative adsorption of H_2_ and D_2_ requires the concurrent segregation of Pd atoms from the subsurface, thus creating a barrier to adsorption that is not present at higher values of *x*_Pd_. This would signify a modification to the 2H′ mechanism that would need to be investigated further. In any case, the presumed inaccessibility of active Pd resulting from Ag saturation at the surface is likely responsible for the increase in Δ*E*_ads_^‡^ from its value of 0 kJ/mol when *x*_Pd_ ≥ 0.64. In addition, the decrease in Δ*E*_ss_ when *x*_Pd_ falls below 0.64 suggests that Pd atoms become enriched in the immediate subsurface of the alloy, making the surface-to-subsurface transition of the adsorbed species more energetically favorable.

### 4.2 Correlation of Activity and Electronic Structure across Ag_*x*_Pd_1–*x*_ Composition Space

Our initial goal was to correlate the kinetic parameters for catalytic H_2_–D_2_ exchange over Ag_*x*_Pd_1–*x*_ alloys with the composition dependent characteristics of their electronic structure. This has been done using computational techniques, like DFT, for several catalytic processes to link the mean energy of the *d*-band, ε̅_*d*_, of various transition metals with the energy barriers, Δ*E*^‡^, for specific elementary steps occurring on their surfaces.^2,56,57^ In this work we have used valence band X-ray photoelectron spectra from a Ag_*x*_Pd_1–*x*_ CSAF to estimate the average energy of the filled valence band center versus composition, ε̅_*v*_ (*x*_Pd_). We have also estimated the kinetic parameters Δ*E*_ads_^‡^, Δ*E*_des_^‡^, and Δ*E*_ss_ versus Ag_*x*_Pd_1–*x*_ alloy composition when applying the 2H′ mechanism for H_2_–D_2_ exchange. However, as discussed in sections 3.4 and 4.1, we cannot conclude with certainty that the kinetic parameters describing H_2_ adsorption, desorption, and surface-to-subsurface diffusion vary appreciably with alloy composition when *x*_Pd_ ≥ 0.64. While ε̅_*v*_ (*x*_Pd_) varies linearly with composition over the range 0.64 ≤ *x*_Pd_ ≤ 1, the energy barriers describing the mechanistic steps for H_2_–D_2_ exchange are possibly all equivalent to the values predicted for pure Pd. This would suggest that the decrease in the activity of the catalysts with decreasing *x*_Pd_ is more strongly tied to the dilution of surface Pd with Ag rather than a change in the electronic structure of the alloy.

Nonetheless, the values of Δ*E*_ads_^‡^, Δ*E*_des_^‡^, and Δ*E*_ss_ predicted by the 2H′ mechanism for H_2_–D_2_ exchange are plotted as a function of the average energy of the valence band, ε̅_*v*_, in Figure S3 of the Supporting Information. Note that these plots are analogous to the plots of Δ*E*_ads_^‡^, Δ*E*_des_^‡^, and Δ*E*_ss_ versus *x*_Pd_ shown in Figure 7 since ε̅_*v*_ is linear in *x*_Pd_ (Figure 4). In this case, the change in catalyst behavior is observed at a threshold of ε̅_*v*_ = −4.5 eV. As ε̅_*v*_ shifts away from the measured valence band energy for pure Pd at ε̅_*v*_^Pd^ = −3.4 eV, Δ*E*_ads_^‡^ remains unchanged at 0 kJ/mol, but suddenly increases to between 15–45 kJ/mol when ε̅_*v*_ < −4.5 eV. On the other hand, shifting away from the Fermi level when ε̅_*v*_ ≥ −4.5 eV results in ∼5 kJ/mol increases in both Δ*E*_des_^‡^ and Δ*E*_ss_ from their values on pure Pd. The kinetic parameter ranges identified previously, Δ*E*_ads_^‡^ = 0–10 kJ/mol, Δ*E*_des_^‡^ = 30–65 kJ/mol, and Δ*E*_ss_ = 20–30 kJ/mol, are valid for up to ∼1 eV shifts below the measured valence band energy for pure Pd. For valence band energy shifts >1 eV below ε̅_*v*_^Pd^ (i.e., when ε̅_*v*_ < −4.5 eV) Δ*E*_ads_^‡^ increases to between 15–45 kJ/mol, the range for Δ*E*_des_^‡^ narrows to between 50–70 kJ/mol, and Δ*E*_ss_ decreases to between 5–20 kJ/mol. The activity of Ag_*x*_Pd_1–*x*_ catalysts for H_2_–D_2_ exchange is negligible when the average valence band energy is less than −4.6 eV.

As shown in Figure 4, the *v*-band energy, ε̅_*v*_, decreases linearly as *x*_Ag_ increases. This decrease in ε̅_*v*_ is accompanied by a decrease in the activity of the Ag_*x*_Pd_1–*x*_ alloy catalysts as seen in Figures 5 and 6. However, despite the apparent relationship between ε̅_*v*_ and catalytic activity, the kinetic parameters for H_2_–D_2_ exchange do not necessarily vary for compositions between 0.64 ≤ *x*_Pd_ ≤ 1. This might initially seem like a surprising result, especially given that some DFT studies have calculated that Δ*E*_ads_^‡^ (ε̅_*d*_) and Δ*E*_des_^‡^ (ε̅_*d*_) decrease as ε̅_*d*_ shifts toward the Fermi level.^57,62−66^ However, since one interpretation of our results suggests that H_2_–D_2_ exchange only occurs on bulk-like Pd domains, it implies that dilution with Ag affects neither the reaction mechanism nor its associated kinetic parameters when *x*_Pd_ ≥ 0.64. It indicates that the decrease in the rate of H_2_–D_2_ exchange with decreasing *x*_Pd_ is not necessarily due to a change in the electronic structure of the alloy, but rather a reduction in the effective surface area accessible for the reaction as the surface becomes Ag saturated. A more direct relationship between the average valence band energy and the kinetic parameters for H_2_–D_2_ exchange might be observed through reduction of the error bars in Figures 7 and S3 by fitting across a larger data set. The step change in the kinetic parameters for Ag_*x*_Pd_1–*x*_ catalysts is observed between −4.6 eV < ε̅_*v*_ < −4.5 eV, where Δ*E*_ads_^‡^ increases and Δ*E*_ss_ decreases relative to the values predicted for Pd. As the valence band energies (and associated Pd compositions) cross this threshold, the heat of adsorption (Δ*E*_ads_^‡^=Δ*E*_ads_^‡^–Δ*E*_des_^‡^) becomes more endothermic due to the increase in Δ*E*_ads_^‡^ while Δ*E*_des_^‡^ remains constant (Figures 7 and S3). Theoretical studies have associated a *d*-band shift away from the Fermi level with increases in Δ*E*_ads_,^62,63,67^ and have similarly linked increases in Δ*E*_ads_ with increases in *x*_Pd_ for Ag_*x*_Pd_1–*x*_ alloy surfaces.^68^ Our findings are consistent with these trends.

It is also worth noting that values of ε̅_*v*_ obtained from XPS measurements in ultrahigh vacuum and from the predictions of computational models fail to capture certain complex processes, such as surface segregation, which are likely at play in our system. Our measurements of the H_2_–D_2_ exchange kinetics across Ag_*x*_Pd_1–*x*_ composition space occurred in an H_2_-rich environment at atmospheric pressure. While surface enrichment of Ag is reported for Ag_*x*_Pd_1–*x*_ alloys under vacuum and inert atmospheres due to its lower surface free energy,^69,70^ inverse surface segregation phenomena have been reported both experimentally^71^ and computationally^72,73^ for Ag_*x*_Pd_1–*x*_ alloys in H_2_-rich atmospheres. In fact, we have recently shown that a Ag_*x*_Pd_1–*x*_ CSAF nearly identical to the one studied here could be activated for ethylene hydrogenation by inducing inverse surface segregation phenomena using trace amounts of a spectator species in the reaction mixture.^5^ The key point to make is that the surface structure and electronic environment of Ag_*x*_Pd_1–*x*_ alloys can be highly variable, resulting in significant changes to catalytic properties under reaction conditions.

## 5 Conclusions

The steady-state H_2_–D_2_ exchange activity of Ag_*x*_Pd_1–*x*_ alloy catalysts was analyzed using reaction data that had been collected using a CSAF and a multichannel microreactor array, enabling kinetic parameter estimation across composition space. The rates of H_2_–D_2_ exchange had been measured at atmospheric pressure, with an order of magnitude change in H_2_ pressure and 2 orders of magnitude change in D_2_ pressure from 333 K – 593 K. The catalytic activity of the CSAF was at its maximum on pure Pd (i.e., *x*_Pd_ = 1) and gradually diminished as *x*_Pd_ decreased until no activity was detected below *x*_Pd_ = 0.58. The 2H′ mechanism for H_2_–D_2_ exchange was fit to the experimental data to estimate its associated kinetic parameters across composition space, and the predictions of the model are consistent with literature expectations for Ag_*x*_Pd_1–*x*_ alloy systems. The ranges Δ*E*_ads_^‡^ = 0–10 kJ/mol, Δ*E*_des_^‡^ = 30–65 kJ/mol, and Δ*E*_ss_ = 20–30 kJ/mol predicted by the 2H′ mechanism for Ag_*x*_Pd_1–*x*_ alloys with *x*_Pd_ ≥ 0.64, suggest that there is a slight kinetic parameter dependence on Pd composition and/or that the reaction only occurs on Pd and not on Ag. Kinetic parameters quite distinct from pure Pd were predicted for alloys with *x*_Pd_ < 0.64. The increase in Δ*E*_ads_^‡^ and decrease in Δ*E*_ss_ observed for active alloys with high Ag content presumably result from the enrichment of Ag at the top surface, restricting accessibility to Pd. XPS measurements of the electronic structure of the Ag_*x*_Pd_1–*x*_ CSAF showed that the average energy of the valence band, ε̅_*v*_, shifted further away from the Fermi level as *x*_Pd_ decreased: decreasing linearly from −3.4 eV for pure Pd to −6.2 eV for pure Ag. Despite the linearity observed for *X*_H2_ (*x*_Pd_) and ε̅_*v*_ (*x*_Pd_), linear relationships between (Δ*E*_ads_^‡^, Δ*E*_des_^‡^, Δ*E*_ss_) and *x*_Pd_ and between (Δ*E*_ads_^‡^, Δ*E*_des_^‡^, Δ*E*_ss_) and ε̅_*v*_ were not observed across the entire range of active alloy compositions. Preferential interactions between H_2_ and Pd likely contribute to these deviations.

## Supporting Information Available

Explicit quantification of the error bars bounding the 95% confidence limits in Figure 7 using the Ag_0.1_Pd_0.9_ catalyst as an example; the criteria for discarding the fitted kinetic parameters at seven active alloy compositions justified using a cutoff value of χ^2^ obtained for the fits; plots of Δ*E*_ads_^‡^, Δ*E*_des_^‡^, and Δ*E*_ss_ versus ε̅_*v*_ (PDF)
