# Supplementary material for: H2–D2 Exchange Activity and Electronic Structure of AgxPd1–x Alloy Catalysts Spanning Composition Space
Source: ACS Catal. 2024 Jul 8;14(14):11014–25. doi: 10.1021/acscatal.4c02309 (PMC11264212; doi:10.1021/acscatal.4c02309)
Supplement: Supplementary file 1 — cs4c02309_si_001.pdf [file cs4c02309_si_001.pdf]

Supporting Information

H_2-D_2 Exchange Activity and Electronic Structure of Ag_xPd_{1-x} Alloy Catalysts Spanning Composition Space

Nicholas Golio[1], Irem Sen[1], Xiaoxiao Yu[1], Petro Kondratyuk[1], and Andrew J Gellman[1,2*]

[1]Department of Chemical Engineering
[2]W.E. Scott Institute for Energy Innovation
Carnegie Mellon University
5000 Forbes Ave., Pittsburgh, PA 15213, United States

*Corresponding author: gellman@cmu.edu, 412-268-3848

1. Quantification of uncertainty for $\Delta E^{\ddagger}_{ads}$, $\Delta E^{\ddagger}_{des}$, and ΔE_{ss}

In order to estimate the uncertainty for $\Delta E^{\ddagger}_{ads}$, $\Delta E^{\ddagger}_{des}$, and ΔE_{ss} at the global minimum for each alloy, the Hessian matrix returned by the solver was used to construct a 3D hyper-ellipsoid bounding the region of 95% confidence around the solution. This 3D hyper-ellipsoid exists in parameter space for $\Delta E^{\ddagger}_{ads}$, $\Delta E^{\ddagger}_{des}$, and ΔE_{ss}, given by ϵ_{ads}, ϵ_{des}, and ϵ_{ss}, respectively, with the optimal solution at the center of the hyper-ellipsoid where χ^2 is at its minimum. Figures S1a-c demonstrate how the uncertainty for the kinetic parameters was calculated for $Ag_{0.1}Pd_{0.9}$. The global minimum in Figures S1a-c is marked by the blue dot at $\Delta E^{\ddagger}_{ads}$ = 0 kJ/mol, $\Delta E^{\ddagger}_{des}$ = 44.8 kJ/mol, and ΔE_{ss} = 26.4 kJ/mol. Grayscale contour maps within parameter space show how ln (χ^2) increases with respect to two kinetic parameters at a time when fixing the third to its value at the global minimum. In other words, Figure S1a shows the behavior of ln (χ^2) with respect to ϵ_{ads} and ϵ_{des} when ΔE_{ss} = 26.4 kJ/mol, Figure S1b shows the behavior of ln (χ^2) with respect to ϵ_{ads} and ϵ_{ss} when $\Delta E^{\ddagger}_{des}$ = 44.8 kJ/mol, and Figure S1c shows the behavior of ln (χ^2) with respect to ϵ_{des} and ϵ_{ss} when $\Delta E^{\ddagger}_{ads}$ = 0 kJ/mol. The red ellipses in Figures S1a-c are 2D cross sections of the 3D hyper-ellipsoid bounding the 95% confidence region when the third parameter is fixed at the global minimum. The red dashed lines trace the constant contour level of ln (χ^2) obtained after performing a Taylor expansion from the global minimum to any point on the red ellipses. Note that the Taylor expansions for Figures S1a-c all yield the same value of ln (χ^2) so the red dashed line in each plot traces the same constant contour level within which any combination of kinetic parameters will yield statistically equivalent solutions with 95% confidence. Since the shape of the contour is difficult to interpret straightforwardly, black lines framing the extrema of the contour level within parameter space were drawn to show where they intersect with the x- and

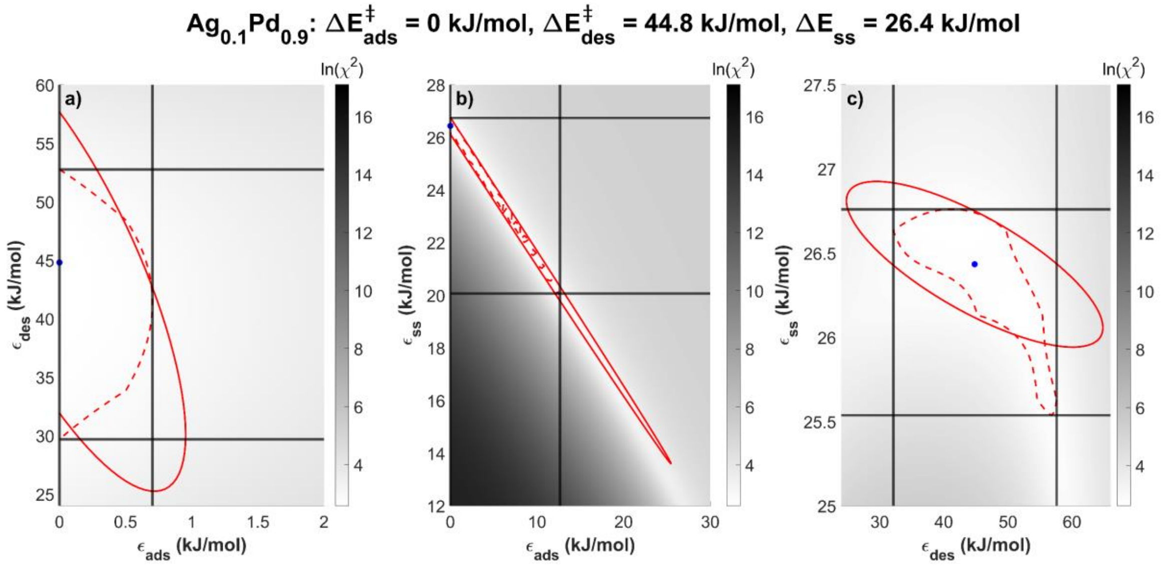

Figure S1. Grayscale map and contour plot of ln (χ^2) within ϵ_{ads}, ϵ_{des}, ϵ_{ss} parameter space plotted around the global minimum for $Ag_{0.1}Pd_{0.9}$ marked by the blue dot at $\Delta E^{\ddagger}_{ads}$ = 0 kJ/mol, $\Delta E^{\ddagger}_{des}$ = 44.8 kJ/mol, and ΔE_{ss} = 26.4 kJ/mol. The grayscale contour maps show how ln (χ^2) increases with respect to two kinetic parameters at a time when fixing the third to its value at the global minimum, i.e., **a)** ΔE_{ss} = 26.4 kJ/mol, **b)** $\Delta E^{\ddagger}_{des}$ = 44.8 kJ/mol, and **c)** $\Delta E^{\ddagger}_{ads}$ = 0 kJ/mol. The Hessian matrix returned by the solver at the global minimum was used to construct a 3D hyper-ellipsoid bounding the region of 95% confidence around the solution. The red ellipses in **a)**, **b)**, and **c)** are the 2D cross sections of the hyper-ellipsoid at the fixed value of the third parameter. The red dashed lines trace the constant contour level of ln (χ^2) obtained by performing a Taylor expansion from the global minimum at χ^2_{min} to any point on the 95% confidence ellipses. Black lines in **a)**, **b)**, and **c)** were drawn to frame the extrema of the red dashed lines within parameter space to show where they intersect with the axes. The 95% confidence region for each kinetic parameter was obtained by defining ranges for $\Delta E^{\ddagger}_{ads}$, $\Delta E^{\ddagger}_{des}$, and ΔE_{ss} that were large enough to include all parameter values within the black lines. For example, while the 95% confidence region for $\Delta E^{\ddagger}_{ads}$ is 0 → 0.7 kJ/mol in **a)**, the region for $\Delta E^{\ddagger}_{ads}$ is 0 → 12.5 kJ/mol in **b)**, and therefore, $\Delta E^{\ddagger}_{ads}$ = 0 → 12.5 kJ/mol was chosen since it includes a larger range and is thus more conservative in defining the 95% confidence region. From **a)**, **b)**, and **c)**, the 95% confidence regions for $Ag_{0.1}Pd_{0.9}$ are given by $\Delta E^{\ddagger}_{ads}$ = 0 → 12.5 kJ/mol, $\Delta E^{\ddagger}_{des}$ = 29.7 → 57.6 kJ/mol, and ΔE_{ss} = 20.1 → 26.8 kJ/mol. These ranges are shown by the error bars for $Ag_{0.1}Pd_{0.9}$ in Figure 7.

y-axis. The 95% confidence region for each kinetic parameter was obtained by defining ranges for $\Delta E^{\ddagger}_{ads}$, $\Delta E^{\ddagger}_{des}$, and ΔE_{ss} that are large enough to include all of the values framed by the black lines. For example, while the 95% confidence region for $\Delta E^{\ddagger}_{ads}$ is 0 → 0.7 kJ/mol in Figure S1a,

the region for $\Delta E^{\ddagger}_{ads}$ is 0 → 12.5 kJ/mol in Figure S1b, and therefore, the larger range was chosen to make the estimate more conservative. Thus, from Figures S1a-c, the 95% confidence regions for the kinetic parameters for $Ag_{0.1}Pd_{0.9}$ are $\Delta E^{\ddagger}_{ads}$ = 0 → 12.5 kJ/mol, $\Delta E^{\ddagger}_{des}$ = 29.7 → 57.6 kJ/mol, and ΔE_{ss} = 20.1 → 26.8 kJ/mol. These ranges are shown by the error bars for $Ag_{0.1}Pd_{0.9}$ in Figure 7. The procedure above was used to estimate the uncertainties for the kinetic parameters predicted at all alloy compositions.

2. Determining outliers for fitted kinetic parameters based on χ^2

Due to inherent noise present in the data, the quality of the fit achieved at each set of optimized kinetic parameters, described by χ^2, varied for each alloy composition. Figure S2a shows χ^2 versus x_{Pd}, and even though there is no obvious trend with respect to Pd content, χ^2 spans an order of magnitude across composition space. While it is clear from the optimization algorithm that solutions with high values of χ^2 have worse fits to the data, it can be difficult to determine the usefulness of the kinetic parameters predicted for each alloy composition without visualizing how the model-predicted values of HD production compare to experimental measurements. Figures S2b-d show the flow rate of HD, F_{HD} (mol/s), versus the reaction temperature, T (K), under reaction conditions where P^{in}_{H2} = P^{in}_{D2} = 230 Torr for three alloy compositions (circled in red in Figure S2a), each with a progressively higher value of χ^2. The alloy composition and value of χ^2 associated with the global minimum are printed on each graph. The black data points in Figures S2b-d are the experimental values of F_{HD} measured at the outlet of the microreactor channels and the red curves show the optimal fits using $\Delta E^{\ddagger}_{ads}$, $\Delta E^{\ddagger}_{des}$, and ΔE_{ss} in the equation for F_{HD} given by the 2H' mechanism for H_2-D_2 exchange. When χ^2 is low, as for $Ag_{0.1}Pd_{0.9}$ in Figure S2b, a good fit to the data is achieved, however, as χ^2 increases, the

quality of the fit diminishes. For example, when χ^2 = 43 in Figure S2c, some discrepancies between the experimental and model-predicted F_{HD} are observed in the middle of the temperature range. Ultimately, when χ^2 increases to 113 in Figure S2d, the model-predicted F_{HD} fails to reproduce the experimental data, as it overfits the data at low T and diverges significantly from experimental measurements for $T \geq 400$ K.

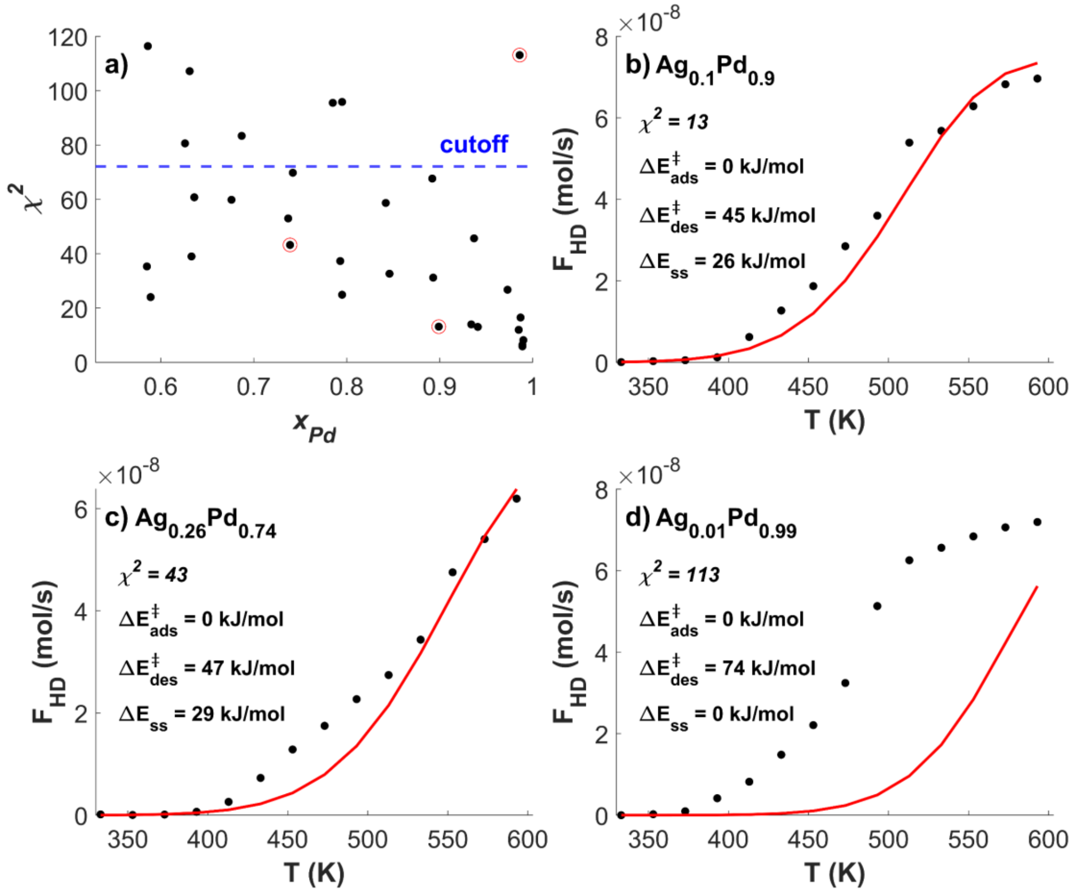

Figure S2. **a)** χ^2 obtained from the best fit kinetic parameters for the 2H' mechanism versus x_{Pd} for $x_{Pd} \geq 0.58$. All data points above the blue dashed line were excluded from Figure 7 due to a poor fit, with $\chi^2 > 1.5 \times \chi^2_{avg}$, where χ^2_{avg} is the average value of χ^2. The data points highlighted by the red circles are shown in **b)**, **c)**, and **d)** where the flow rate of HD, F_{HD} (mol/s), is plotted versus the reaction temperature, T (K), when P^{in}_{H2} = P^{in}_{D2} = 230 Torr for $Ag_{0.1}Pd_{0.9}$, $Ag_{0.26}Pd_{0.74}$, and $Ag_{0.01}Pd_{0.99}$, respectively. The black points are the experimental values of F_{HD} measured at the outlet of the microreactor channels and the red curves show the model-predicted F_{HD} using the optimal kinetic parameters. When χ^2 is low (i.e., χ^2 = 13 in **b)**), a good fit to the data is achieved, however, as χ^2 increases (i.e., χ^2 = 43 in **c)**), the quality of the fit diminishes until it ultimately fails to capture the correct trend when χ^2 becomes too high (i.e., χ^2 = 113 in **d)**). The deterioration of the fit from **b)** to **c)** to **d)** shows progressive overfitting to low temperature data as χ^2 increases, making a bad overall prediction for F_{HD}. Consequently, the kinetic parameters corresponding to these predictions must be omitted from subsequent analysis since they do not appropriately characterize the data set. The cutoff value of χ^2 was chosen to be $1.5 \times \chi^2_{avg}$ (the blue dashed line in **a)**) based upon the point at which visual analysis of F_{HD} versus T shows a failure to fit the data properly.

It is important to note at this point that the data at P_{H2}^{in} = P_{D2}^{in} = 230 shown in Figures S2b-d are only a subset of the entire set of F_{HD} measurements used for the fitting. There are 13 other combinations of P_{H2}^{in} and P_{D2}^{in} for which F_{HD} versus T can be plotted using the optimal kinetic parameters and it is the total sum of squared errors from these 196 data points that was used to calculate the value of χ^2 shown in Figures S2b-d. The key takeaway is that the quality of the fit to F_{HD} shown in Figures S2b-d is similar at other combinations of P_{H2}^{in} and P_{D2}^{in} across the entire data set. This highlights the fact that even though the solver was able to converge on a global minimum within parameter space, this does not inherently mean that the estimated values of $\Delta E_{ads}^{\ddagger}$, $\Delta E_{des}^{\ddagger}$, and ΔE_{ss} are useful for describing H_2-D_2 exchange.

Based upon visual analysis of the fits of F_{HD} versus T at different values of χ^2, it was determined that an appropriate cutoff value for χ^2 was 1.5 × χ_{avg}^2, where χ_{avg}^2 is the average value of χ^2 across all data points. The cutoff value of χ^2 = 72.1 is marked by the blue dashed line in Figure S2a. All values of χ^2 below the cutoff correspond to global minima where the estimated kinetic parameters provide useful estimates for F_{HD} across all T, P_{H2}^{in}, and P_{D2}^{in}. On the other hand, the 7 alloys having values of χ^2 above the cutoff correspond to solutions where there is significant overfitting to F_{HD} at low T (as in Figure S2d), leading to poor F_{HD} predictions across most reaction temperatures and all inlet pressure combinations. These solutions are outliers since they do not properly characterize H_2-D_2 exchange on Ag_xPd_{1-x} alloy catalysts. Consequently, the kinetic parameters corresponding to these improper fits were removed from Figure 7 and were not included in the analysis of $\Delta E_{ads}^{\ddagger}$, $\Delta E_{des}^{\ddagger}$, and ΔE_{ss} as a function of x_{Pd} and $\bar{\varepsilon}_v$.

3. Kinetic parameters $\Delta E_{ads}^{\ddagger}$, $\Delta E_{des}^{\ddagger}$, and ΔE_{ss} versus $\bar{\varepsilon}_v$

The kinetic parameters for H_2-D_2 exchange predicted by the 2H' mechanism, $\Delta E^{\ddagger}_{ads}$, $\Delta E^{\ddagger}_{des}$, and ΔE_{ss}, are plotted as a function of the average energy of the valence band, $\bar{\varepsilon}_v$, in Figure S3. Figure S3 is analogous to Figure 7 showing $\Delta E^{\ddagger}_{ads}$, $\Delta E^{\ddagger}_{des}$, and ΔE_{ss} versus x_{Pd} since $\bar{\varepsilon}_v$ is linear in x_{Pd}, as shown in Figure 4. As $\bar{\varepsilon}_v$ shifts away from the Fermi level, $\Delta E^{\ddagger}_{ads}$ remains unchanged

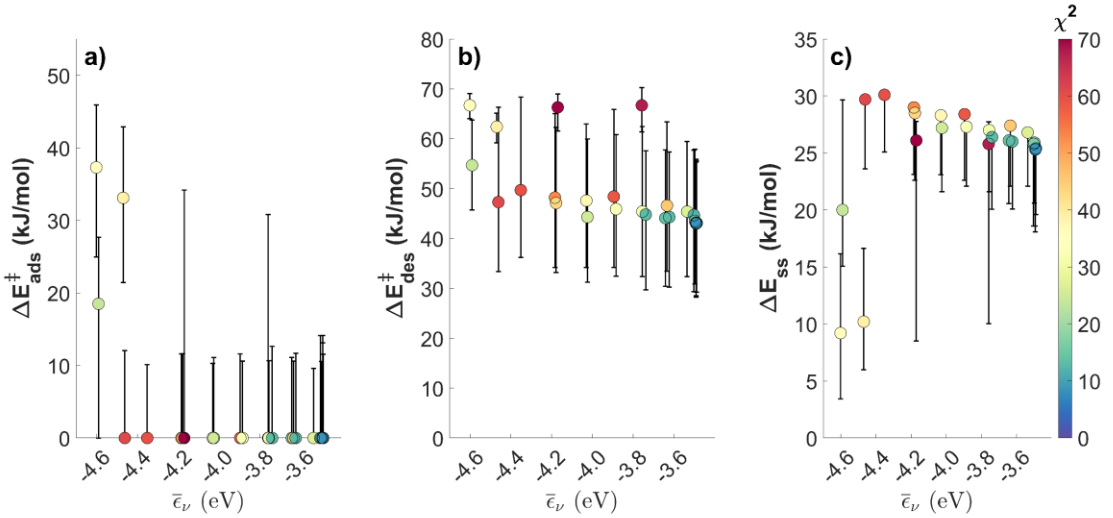

Figure S3. Kinetic parameters for H_2-D_2 exchange predicted by the 2H' mechanism, **a)** $\Delta E^{\ddagger}_{ads}$, **b)**, $\Delta E^{\ddagger}_{des}$, and **c)** ΔE_{ss}, versus the average energy of the v-band, $\bar{\varepsilon}_v$. This figure is analogous to Figure 7 showing the kinetic parameters versus x_{Pd} since $\bar{\varepsilon}_v$ is linear in x_{Pd} (Figure 4). The value of χ^2 for the fitted kinetic parameters is indicated by the color of the data point and corresponds to the color scale at the right of the figure. As $\bar{\varepsilon}_v$ shifts away from the Fermi level, $\Delta E^{\ddagger}_{ads}$ remains unchanged at 0 kJ/mol when $\bar{\varepsilon}_v \geq$ -4.5 eV, but suddenly increases to between 15-45 kJ/mol when $\bar{\varepsilon}_v <$ -4.5 eV. On the other hand, when $\bar{\varepsilon}_v \geq$ -4.5 eV, $\Delta E^{\ddagger}_{des}$ and ΔE_{ss} appear to increase by ~5 kJ/mol as $\bar{\varepsilon}_v$ shifts away from the Fermi level. However, as before, the error bars representing the 95% confidence limits around the global minima prevent us from confirming that these trends are not merely artifacts resulting from the coupling of the kinetic parameters in the 2H' mechanism. Consequently, the conservative kinetic parameter ranges are $\Delta E^{\ddagger}_{ads}$ = 0-10 kJ/mol, $\Delta E^{\ddagger}_{des}$ = 30-65 kJ/mol, and ΔE_{ss} = 20-30 kJ/mol for up to ~1 eV shifts below the measured valence band energy for pure Pd, $\bar{\varepsilon}_v^{Pd}$ = -3.4 eV. A sudden change in the kinetic parameters is observed when $\bar{\varepsilon}_v <$ -4.5 eV, where $\Delta E^{\ddagger}_{ads}$ increases to between 15-45 kJ/mol, the range for $\Delta E^{\ddagger}_{des}$ narrows to between 50-70 kJ/mol, and ΔE_{ss} decreases to between 5-20 kJ/mol.

at 0 kJ/mol when $\bar{\varepsilon}_v \geq$ -4.5 eV, but suddenly increases to between 15-45 kJ/mol when $\bar{\varepsilon}_v <$ -4.5 eV. On the other hand, when $\bar{\varepsilon}_v \geq$ -4.5 eV, $\Delta E^{\ddagger}_{des}$ and ΔE_{ss} appear to increase by ~5 kJ/mol as $\bar{\varepsilon}_v$ shifts away from the Fermi level. However, as before, the error bars representing the 95% confidence limits around the global minima prevent us from confirming that these trends are not merely artifacts stemming from the coupling of the kinetic parameters in the 2H' mechanism. Consequently, the conservative kinetic parameter ranges are $\Delta E^{\ddagger}_{ads}$ = 0-10 kJ/mol, $\Delta E^{\ddagger}_{des}$ = 30-65 kJ/mol, and ΔE_{ss} = 20-30 kJ/mol for up to ~1 eV shifts below the measured valence band energy for pure Pd, $\bar{\varepsilon}_v^{Pd}$ = -3.4 eV. A sudden change in the kinetic parameters is observed when $\bar{\varepsilon}_v <$ -4.5 eV, where $\Delta E^{\ddagger}_{ads}$ increases to between 15-45 kJ/mol, the range for $\Delta E^{\ddagger}_{des}$ narrows to between 50-70 kJ/mol, and ΔE_{ss} decreases to between 5-20 kJ/mol. The activity of Ag_xPd_{1-x} catalysts for H_2-D_2 exchange is negligible when the average valence band energy is less than -4.6 eV.
